# Evaluation of OCT biomarker changes in treatment-naive neovascular AMD using a deep semantic segmentation algorithm

**DOI:** 10.1038/s41433-024-03264-1

**Published:** 2024-07-27

**Authors:** Ben Asani, Olle Holmberg, Johannes B. Schiefelbein, Michael Hafner, Tina Herold, Hannah Spitzer, Jakob Siedlecki, Christoph Kern, Karsten U. Kortuem, Amit Frishberg, Fabian J. Theis, Siegfried G. Priglinger

**Affiliations:** 1 Department of Ophthalmology, Ludwig-Maximilians-University, Munich, Germany; 2 Institute of Computational Biology, Helmholtz Centre Munich, Munich, Germany; 3 Department of Ophthalmology, Ulm University, Ulm, Germany; 4 Department of Mathematics, TU Munich, Munich, Germany

**Keywords:** Tomography, Predictive markers, Retinal diseases

## Abstract

**Objectives:**

To determine real-life quantitative changes in OCT biomarkers in a large set of treatment naive patients in a real-life setting undergoing anti-VEGF therapy. For this purpose, we devised a novel deep learning based semantic segmentation algorithm providing the first benchmark results for automatic segmentation of 11 OCT features including biomarkers for neovascular age-related macular degeneration (nAMD).

**Methods:**

Training of a Deep U-net based semantic segmentation ensemble algorithm for state-of-the-art semantic segmentation performance which was used to analyze OCT features prior to, after 3 and 12 months of anti-VEGF therapy.

**Results:**

High F1 scores of almost 1.0 for neurosensory retina and subretinal fluid on a separate hold-out test set with unseen patients. The algorithm performed worse for subretinal hyperreflective material and fibrovascular PED, on par with drusenoid PED, and better in segmenting fibrosis. In the evaluation of treatment naive OCT scans, significant changes occurred for intraretinal fluid (mean: 0.03 µm^3^ to 0.01 µm^3^, *p* < 0.001), subretinal fluid (0.08 µm^3^ to 0.01 µm^3^, *p* < 0.001), subretinal hyperreflective material (0.02 µm^3^ to 0.01 µm^3^, *p* < 0.001), fibrovascular PED (0.12 µm^3^ to 0.09 µm^3^, *p* = 0.02) and central retinal thickness C0 (225.78 µm^3^ to 169.40 µm^3^). The amounts of intraretinal fluid, fibrovascular PED, and ERM were predictive of poor outcome.

**Conclusions:**

The segmentation algorithm allows efficient volumetric analysis of OCT scans. Anti-VEGF provokes most potent changes in the first 3 months while a gradual loss of RPE hints at a progressing decline of visual acuity. Additional research is required to understand how these accurate OCT predictions can be leveraged for a personalized therapy regimen.

## Introduction

Ocular coherence tomography (OCT) imaging of the retina and the identification of several disease biomarkers has become key in creating treatment protocols and tracking of disease progression in neovascular age-related macular degeneration (nAMD) [1]. Today, decision for treatment is mainly guided by the findings in OCT images of nAMD patients where several biomarkers have been detected to guide therapeutic decisions [2–5]. That has, in the past, led to an overall high therapy intensity with monthly to bimonthly injections over several years, posing a major challenge for both patients and the healthcare system: A recent study by Chopra et al. shows an 11-fold increase in annual intravitreal injections from 2009 to 2019 and is projected to continue to rise [6]. Newer studies, however, have shown that ignoring subretinal fluid (SRF) (<200 µm at the foveal center) does not change outcome in visual acuity for patients but can lessen their treatment burden [7]. Additionally, a very stable fibrovascular pigment epithelial detachment (fPED) may correlate with a protection for the development of macular atrophy [8].

Consequently, to understand the prognostic value of different biomarkers it is important to assess their overall distribution in patients in a real-life setting under optimal therapeutic adherence. However, manually analyzing these and their changes over time becomes difficult as well as time consuming once we want to study a greater set of patients. Additionally, the variety of morphologic features in AMD add much to the complexity and heterogeneity of the scan [9]. This generates high-volume data making the manual segmentation process close to impossible.

In this project, we propose a deep learning-based semantic segmentation algorithm trained with 458 manually annotated macular OCT scans, to allow automatic segmentation of clinical features of a large series of treatment-naive patients eyes suffering from neovascular AMD and undergoing anti-VEGF (vascular endothelial growth factor) treatment.

We additionally show a validation of the algorithm on independent test sets of previously unseen patients combined with detailed analysis of the inter-annotator variance for ambiguous and hard to annotate features. It furthermore allows us to give a one-of-a-kind extensive description on the distribution of morphologic features as well as disease biomarkers of that patient group found in a real-life setting.

## Methods

This case series included patients from our clinic with treatment-naive nAMD in the study eye and had a follow-up observational period of at least three and twelve months. Treatment naive was defined as never having had any form of intravitreal injection. with the first injection received at time of diagnosis. Exclusion criteria were comorbidities such as central retinal vein occlusion, retinal branch occlusion, diabetic macular edema, uveitis, and other conditions that can lead to the development of intra- or subretinal fluid. The study was approved by the institutional review board of our institution and adhered to the tenets of the Declaration of Helsinki. Written informed consent was obtained from each participant prior to the intervention and all testing outlined herein.

### Treatment regimen

Patients received an upload of three monthly injections of any of anti-VEGF (Ranibizumab, Aflibercept, or Bevacizumab) and were then treated according to the Treat and Extend regimen: They were either extended for two weeks or continued on a monthly injection routine [10].

### Patient identification

We queried our data warehouse for all patients receiving intravitreal Injections of anti-VEGF between 2013/03/11 and 2020/07/09. Diagnosis of neovascular AMD was confirmed after proof of choroidal neovascularization in initial Fluorescein Angiography. We interpolated the data set to 3 measurement points: Start date is the time of the first intravitreal injection. Next monitoring point is after 3 months and lastly after 12 months of treatment.

### Preoperative examinations

Examinations before intravitreal injections included testing best corrected visual acuity (BCVA) using standard Snellen Chart, intraocular pressure using non-contact tonometry, dilated indirect fundoscopy as well as spectral-domain optical coherence tomography of the macula (Spectralis; Heidelberg Engineering GmbH, Heidelberg, Germany). The metrics “counting fingers”, “hand movement” were converted to 1.98 and 2.28 logMar respectively as previously described by Lange et al. and Schulze-Bonsel et al. [11, 12]. All visual acuity values in this study are given in logMAR units.

### Segmentation data sets

To create the segmentation algorithm, a set of 458 macular OCT scans, each from a different patient, were annotated by fellows in medical retina (B.A., J.B.S., and M.H.) using the annotation tool LabelMe [13]. They were then validated by three retinal experts (J.S., C.K., and T.H.) and re-labeled in case of any discrepancies. Each fellow was assigned his or her own set randomly. In this process, we followed the Consensus Nomenclature for Reporting Neovascular Age-Related Macular Degeneration of the AAO (American Academy of Ophthalmology) for disease biomarkers and used 11 different OCT labels [14]. The annotation was made pixel wise, i.e., each pixel in the image was assigned one of the 11 classes: Epiretinal Membrane (ERM), Neurosensory Retina (NR), Retinal pigment epithelium (RPE), Intraretinal fluid (IRF), SRF, Subretinal hyperreflective material (SHRM), Drusenoid pigment epithelial detachment (dPED), fPED, Fibrosis, Choroid, Posterior hyaloid membrane (PHM). For annotation examples as well as exact class distribution statistics see supplement. The central retinal thickness C0 was defined according to the ETDRS grid [15].

An additional data set of 30 scans were annotated to measure the inter-annotator variation between the three annotators for a selection of ambiguous features. To quantify feature ambiguity as well as establishing an upper bound for how well these features can be expected to be segmented, the ophthalmological fellows all annotated the same scans. Afterwards, a consensus annotation was produced by a panel including the retinal experts producing in total four annotations for each of the 30 scans.

### Model architecture

The algorithm used for segmenting the retinal OCT scans is a deep convolutional neural network [16] of a U-net type architecture [17]. Specifically, the network consists of eleven convolution layers followed by batch normalization [18] and relu activation functions [19]. The convolutional layers use padding so as to not alter the dimensions of the feature maps and have kernel size set to three throughout the network. Each convolutional layer is initialized using the He normal initialization [20] at the start of the training. In the encoder, every two convolutional layers are followed by a max pooling operation making a total of five max poolings, reducing the resolution size of the input from 256 to 8 for the feature maps. Here, the original images are linearly resized from 512 to 256 pixels height and width. The first convolutional layer is set to have 64 filters and this number is doubled after every max pooling layer yielding a maximum of 1024 filters in the bottleneck of the architecture. The number of filters are then halved after every transposed convolution in the decoder. Between the encoder and the decoder a dropout layer with probability 0.2 is applied for regularization. In the decoder, transposed convolutions as well as two layered convolutional blocks as described above are applied consecutively until the original input dimension is reached. After filtering and max pooling operations a convolutional layer with kernel size one and a softmax activation function is applied to achieve the final output of the network.

### Model training

The models were trained using the Adam optimizer using the categorical cross-entropy loss, with an initial learning rate of 0.001, found to be optimal through hyperparameter tuning on a validation set. The images and annotation masks were split into a train, validation, and test set consisting of 338, 84, and 36 images, respectively. Further, an ensemble of networks was created for each image in the 36-image test set using a leave-one-out validation scheme and adding the remaining test images to the training data set. In total five models were trained for each test image resulting in 180 different models. At inference time, the softmax outputs for each class and pixel, from all five models, were averaged to obtain an ensemble prediction. The class with the highest average softmax score yielded the final prediction. For the inter-doctor variance data set the 10 models, out of 180, with the best validation scores were selected to form an ensemble from which the predictions were obtained in the same way as for the test set.

### Model evaluation

The segmentation model is evaluated using the F1 score, i.e., the harmonic mean between precision and recall, a standard evaluation metric for semantic segmentation tasks. The score is then presented for each class. Further the inter-doctor variance is presented as the F1 score between each annotator and the 10-model ensemble against the consensus annotation. As all pixels are concatenated for all images, as typically done in semantic segmentation tasks, no standard deviation metrics between images are provided. The statistically evaluated model was then used for automatic segmentation of 18,522 OCT scans from 378 eyes enabling the statistical analysis of morphological OCT features including nAMD biomarker distribution on treatment naive patients and how they are affected by anti-VEGF injections.

### Statistical analysis

All statistical analysis was performed using the Python programming language with the Scipy stats software package [21]. Normality of data was assumed due to sufficiently large sample sizes well above 30 as specified by the central limit theorem [22]. We applied the independent samples *t*-test for parametric comparisons. The level of statistical significance was defined as *p* < 0.05.

The code for the models and training procedures as well as result analysis will be made available through the public Github repository upon publication.

## Results

### Deep learning segmentations accurately quantifies presence of clinical features in retinal OCT images

**Fig. 1:**
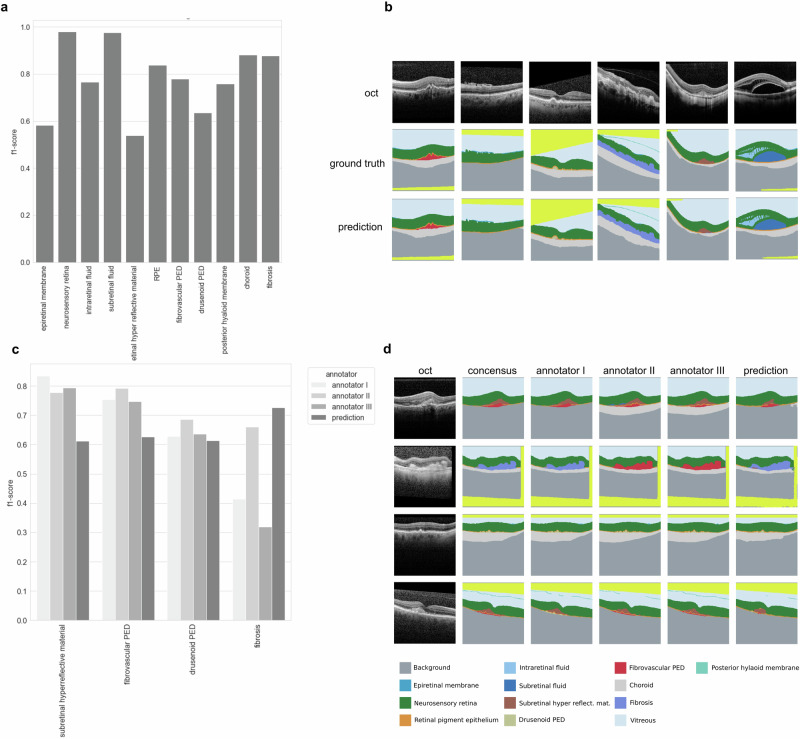
Deep learning segments clinical features on par with human experts from retinal OCT images. **a** F1 scores for 11 clinical features segmented on a test set from 37 to the algorithm previously unseen patients. **b** Example of OCT images selected to illustrate various segmentation classes with ground truth and predicted segmentation maps. **c** F1 scores for subretinal hyperreflective material, fibrovascular PED, drusenoid PED as well as fibrosis on 30 challenging test patients containing these features. **d** Example OCT images with consensus ground truth, annotations from three different annotators as well as predicted segmentation maps displaying segmentation of multiple features. Yellowish color was not evaluated and either stands for a crop of the image or vitreous cavity.

The clinical features were segmented with a top performance of 0.98 F1-score for features NR and SRF. The lowest F1-scores were observed for features ERM, dPED, and SHRM (see Fig. 1a). Test set examples of the segmented features can be seen in Fig. 1b, showing the variety of features accurately segmented in unseen patients. The annotators, on the other end, highly agreed on SHRM, fPED, and dPED, while largely disagreeing in the case of fibrosis (see Fig. 1c). Overall, the segmentation algorithm performed worse than the annotators on SHRM and fPED, on par with respect to dPED and better than all annotators when segmenting fibrosis (see Fig. 1c). In Fig. 1d we see examples of consensus, annotator and segmentation algorithm predicted segmentations for example OCT images.

### Most morphological changes of treatment naive patients occur during the first three months of anti-VEGF therapy

**Fig. 2:**
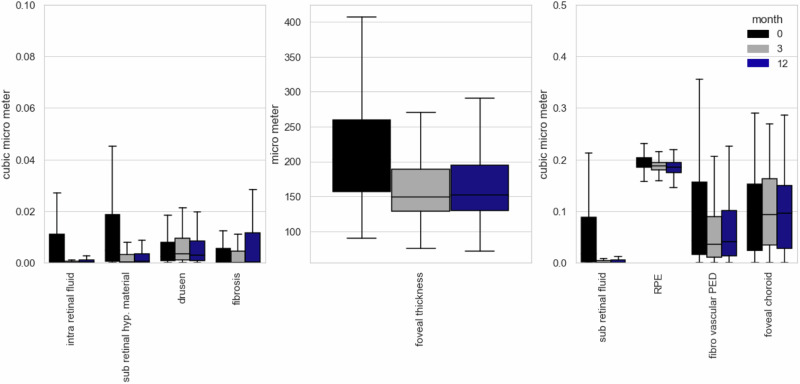
Volumetric changes of treatment naive OCT biomarkers under anti-VEGF treatment. Significant reduction was seen in IRF, SRF, SHRM, RPE and central foveal thickness.

After filtering for the inclusion criteria a total of 378 eyes consisting of 18,522 OCT scans from 339 different patients were segmented and analyzed. Of those patients 144 were male and 234 female with an average age of 82 ± 8 years. Features in treatment-naive patients that were most prominent on initial presentation were by far fPED (Mean: 0.12 µm^3^, SD: 0.19 µm^3^), followed by SRF (Mean: 0.08 µm^3^, SD: 0.26 µm^3^), IRF (Mean 0.03 µm^3^, SD 0.08 µm^3^) and SHRM (Mean: 0.02 µm^3^, SD: 0.05 µm^3^, see Fig. 2). Mean number of injections was 3.8 ± 1.5 after 3 months and 8.3 ± 3.5 after 12 months, meaning that on average the therapy regimen was extended at some point during the observed time frame.

After 3 months of intravitreal treatment there was little but significant change in fPED from 0.12 µm down to 0.09 µm (SD: 0.15 µm, *p*-value = 0.02) but no difference afterwards. The overall change of this marker over the course of twelve months was barely not statistically significant (*p*-value: 0.05) meaning that therapy in general did not lead to real regression of the fPED but seems to halt further growth.

Difference in means as tested by t-tests showed a statistical significant change in IRF, SRF, SRHM, and CRT C0 after 3 months of anti-VEGF therapy but no significant improvement or change until month 12 occurred, meaning setbacks under therapy were uncommon. This was on par with the observed changes in visual acuity. Significant improvement was achieved from before treatment during the first 3 months (mean VA on month 1: 0.59 logMAR, mean VA on month 3: 0.50 logMAR; *p*-value: 0.003) but no significant change occurred afterwards up until month 12. The distribution of these and the other morphological features including disease biomarker amongst treatment naive patients is summarized in Fig. 2 and Table 1. The RPE showed a significant decline throughout the complete observation period therefore hinting at a steady increase in atrophy.

**Table 1:** Mean volumetric values before (mean 1), three months (mean 3) and twelve months after initiation of anti-VEGF therapy.

	ERM	IRF	SRF	SRHM	RPE	fvped	drusen	PHM	Choroid	Fibrosis	CRT
Mean 1	0·01	0·03	0·08	0·02	0·19	0·12	0·01	0·02	0·93	0·02	223·82
Mean 3	0·01	0·01	0·01	0·01	0·19	0·09	0·01	0·02	0·96	0·02	169·65
Mean 12	0·01	0·01	0·01	0·00	0·18	0·09	0·01	0·02	0·95	0·03	172·62
sd 1	0·02	0·08	0·25	0·05	0·02	0·18	0·01	0·03	0·41	0·06	88.29
sd 3	0·02	0·07	0·09	0·02	0·02	0·15	0·01	0·03	0·41	0·06	64·11
sd 12	0·02	0·04	0·04	0·01	0·02	0·15	0·01	0·03	0·41	0·08	63·76
*p*-value (1–3)	0·62	0·00*	0·00*	0·00*	0·00*	0·02	0·44	0·39	0·33	0·59	0·00*
*p*-value (1–12)	0·38	0·00*	0·00*	0·00*	0·00*	0·05	0·82	0·24	0·50	0·15	0·00*
*p*-value (3–12)	0·70	0·94	0·86	0·32	0·01	0·59	0·30	0·74	0·76	0·05	0·52

*p < 0.001.

All values are in µm^3^.

We additionally used linear regression to model the interdependence between the different biomarkers and visual acuity outcome in 12 months. There was a negative correlation between visual acuity and intraretinal fluid, ERM, fPED, and fibrosis. Since most of these features are most prominent in the active disease state (excluding ERM) and bring immense structural changes to the retina, this finding does not seem very surprising. Interestingly, SRF had no correlation with a worse outcome in visual acuity, supporting the thesis of being protective against retinal atrophy and further decline of vision. Other findings are summarized in Table 2.

**Table 2:** Results from multiple regression analysis of oct markers and their effect on 12 month visual acuity (*n* = 336).

Model predictors	*b*	SE b	*t*	*p*
Intercept	0.64*	0.23	2.82	0.01
ERM	2.5**	0.90	2.51	0.01
IRF	1.24***	0.29	4.32	0.00
SRF	−0.15	0.09	−1.73	0.08
SRHM	0.64	0.47	1.35	0.18
RPE	−1.58	1.25	−1.26	0.21
fvped	0.37*	0.14	2.64	0.01
drusen	−1.19	2.06	−0.58	0.56
PHM	−0.36	0.57	−0.63	0.53
Choroid	0.02	0.05	0.55	0.59
Fibrosis	1.03	0.52	0.05	0.05
crt	0.17	0.38	0.65	0.65
Adj. *R*^2^	0.189			
*F*	8.08***			

*Note*. *p < 0.05, **p < 0.01, ***p < 0.001.

## Discussion

Structural changes in OCT images of nAMD patients have been extensively studied in the past [5, 23] however, not to the extent that is made possible by a high-performing segmentation algorithm. In this work, we were able to realistically describe not only the morphologic characteristics of the patients but also actually quantify those volumetrically including the disease biomarkers in their respective scans. This was leveraged to give a one-of-a-kind description of a large number of treatment-naive nAMD patients in a real-life setting.

### Contribution to the application of deep learning algorithms in understanding the retina

Semantic segmentation of OCT images has been studied before [24–27]. A similar segmentation algorithm was developed by De Fauw and colleagues [28] which also uses a segmentation network as a pre-processing method for predicting retinal disease. Our work differs with regards to two main aspects. First, we include the latest consensus nomenclature as specified by the AAO, and secondly, we provide an in-depth analysis of feature ambiguity by inter-annotator variation analysis and test performance metrics reporting on a large number of unseen test patients. This entails evaluating uncertainties surrounding feature ambiguities by examining how various retinal experts interpret different image features through annotation comparisons and then testing the algorithm’s performance against these experts to assess its accuracy. We show that segmentation of the above features is indeed possible to a high degree of accuracy (see Fig. 1a). While the algorithm segments at a similar proficiency as the annotators and even outperforms the retinal fellows at segmenting fibrosis, we also clearly show the current limitation of segmentation of the retina by quantifying the ambiguity of the features fibrosis, drusen, and SHRM (see Fig. 2).

These difficulties specifically present in transitions from fPED to fibrosis as they would appear very ambiguous on OCT scans. For example, Ohayon et al. segmented fibrovascular PED into three layers with a more hyperreflective layer 2 that did not respond as efficiently as the other layers following anti-VEGF treatment being suggestive of a fibrotic component in the PED [29]. Interestingly, in accordance to that, the segmentation algorithm presented in this study occasionally segmented the fibrovascular PED into a fibrotic sub-compartment as well, making the segmentation process in this case potentially more accurate than the training set provided by the retinal fellows as they had to make a definite decision on which biomarker was present for the whole structure. However, this warrants further investigation. Lastly, due to the lower quality in some of the scans, it seemed difficult to precisely isolate the borders between the different OCT classes on a pixel-wise level.

Another particular issue with algorithms trained on a specific training set is the transferability to different devices with possibly other axial resolutions. However, as shown in a previous study, we demonstrated that this is indeed possible [30]. SVDNA, a minimal method for unsupervised domain adaptation, shows the transferability of this algorithm to other devices such as Dri-OCT (Topcon, Tokyo, Japan), Cirrus OCT (Carl Zeiss AG, Oberkochen, Germany), and Bioptigen-OCT (Leica, Wetzlar, Germany). We show that it performs at least en par or even outperforms the more complex state-of-the-art UDA methods while considerably reducing training complexity [30].

### Distribution of biomarkers and their overall plasticity on treatment-naive patients

We show that the most prominent change was to be seen in the first three months suggesting the initial upload phase of three injections as being the most potent for morphological changes to the retina. Interestingly, almost no changes could be determined afterwards until month 12, revealing that any further injection past the initial monthly upload phase seems to overwhelmingly function as a stabilizing agent. The biomarkers most sensitive to change were usually the ones that have been most prominent at the beginning: IRF, SRF, SHRM, RPE, and fPED.

We showed that of those markers only IRF and fPED were predictive of a generally worse visual acuity outcome after 12 months. Although it seems logical that higher amounts indicate higher disease activity, we couldn’t find any prior evidence that would necessarily link the actual volumetric amount of fluid to a different outcome in contrast to fPED for which there was prior evidence for example as described by Boyer et al. [31]. One reason would be that patients with an abundance of these markers might have generally presented later after the first onset of symptoms and thus natural progression of the disease would have led to more irreversible retinal damages as delay to treatment is a significant factor for a poorer outcome [32]. Other than these, fibrosis and ERM correlated negatively with visual acuity which is in direct contradiction to earlier findings by Alkin et al., where they concluded comparable results (with and without ERM) after treatment with bevacizumab [33].

Our findings also clearly suggest a gradual loss of RPE throughout 12 months. This might suggest a relationship between intravitreal anti-VEGF and retinal atrophy as it has been proposed in the past. Direct proof is still missing [34] however, and the loss was happening unaffected by change in injection intensity and favors the thesis of a natural disease progression.

Similar descriptive studies have been done in the past but with a much smaller case series, manual segmentation and less biomarkers which in general led to various findings [29, 31, 35, 36].

Lai et al. observed treatment response for intraretinal cysts (IRC), SRF, PED, and their correlation with BCVA changes for a time period of a year whilst setting similar time points as in our study (months 1, 3, 6, and 12) [35]. In 126 eyes only 33.3% showed a resolution of their PED whereas IRC resolved in 53.8% and SRF in 51.6% of cases [35]. In correlation with that we saw a statistically significant and prominent volumetric reduction in IRF and SRF, whilst the decrease in fPED was not significant over a period of 12 months. Other observations by Golbaz et al. demonstrated that IRF and SRF immediately responded to anti-VEGF treatment albeit the overall plasticity of the morphological changes declined over time which corroborates with our findings. Interestingly the sub-RPE compartments showed the least or no changes to anti VEGF therapy [36]. The high immediate effect of anti-VEGF on especially IRF or SRF is all in all well documented in several other studies in the literature [37]. Bolz et al. for example showed a significant effect of anti-VEGF on retinal fluid compartments as early as one week after the injection whilst there was no significant change after the second and third injection [38]. While this might be suggestive of cutting the loading dose to just one injection, the small number of cases (*n* = 29) and missing long-term observation of relapsing cases in that study warrants caution.

None of the aforementioned studies documented any changes to the RPE layer nor did we find any other studies observing a steady loss in RPE with nAMD patients under anti-VEGF therapy, at least not in a large cohort as in the treatment-naive dataset presented here.

Our study is mainly limited by its retrospective nature. However, given the high number of real treatment-naive patients in this study, the findings can more or less be very suggestive on the effect of anti-VEGF treatment. Moreover, it gives us a general idea on a broad population of patients, and what morphological properties they present upon first presentation. Other limitations include the discussed feature ambiguities and the lower F1 scores for some of the biomarkers. While this could lead to some changes being misinterpreted, the F1 numbers are still quite high in general. In some cases, they can be artificially lower as for certain features (especially membranes) it is difficult for manual annotators to segment correctly down to a pixel level with the precision that an automatic segmentation algorithm would. These micro-differences negatively impact F1 scores, albeit the algorithm being able to handle real segmentation of these features smoothly.

To conclude, our study shows the feasibility of a high-performing segmentation algorithm to quantitatively segment the whole region of a much larger number of OCT volume scans and thereby enable precise determination of volumetric changes down to a microscopic level. With that we were able to present a general description of the volumetric distribution of biomarkers in treatment-naive patients and confirm that the most significant plasticity in biomarkers happens during the first 3 months of therapy with changes coming to a halt afterwards besides a gradual loss of the RPE layer which seems to continue at least until the end of the observed time frame. For future work, this algorithm and findings could be used to analyze larger time series data sets and possibly predict the best-fitting therapy for each individual patient leading the way to a more personalized approach in treatment regimens.

## Summary

### What was known before

- Limitations in Algorithmic Research Quality and Biomarker Inclusion: Existing segmentation algorithms to date present substantial quality ambiguities and lack consistent up-to-date biomarkers. Furthermore, a validation on independent test sets seems to be absent, which represents a notable gap in the current state of OCT segmentation research, raising questions about their reliability and generalizability.
- Shortcomings in characterization of treatment naive AMD While existing literature, encompassing trials like MARINA, VIEW, or HORIZON, provide a comprehensive understanding of age-related macular degeneration (AMD) as a whole, the characterization of treatment-naive AMD patients, including detailed volumetric analysis of retinal morphology and disease biomarkers, remains absent. Studies focusing on treatment-naive patients typically exhibited limitations such as small sample sizes, a constrained array of biomarkers, and incomplete volumetric data, which hindered the ability to gain a holistic understanding of this patient group.

### What this study adds

- Improving existing segmentation algorithms for big data analysis: This study improves segmentation algorithms in a safe and high quality manner as it is tested on independent test sets which then can be leveraged for big data analysis to define risk factors in the process. Furthermore, it follows the Consensus Nomenclature for Reporting Neovascular Age-Related Macular Degeneration of the AAO for disease biomarkers.
- Extensive descriptive analysis of a large amount of treatment-naive nAMD patients: The analysis of a large cohort of treatment-naive patients in a real-world setting as well as analysis of changes in the various OCT biomarkers over time and under therapy gives us a more precise anatomical insight to the disease while defining risk factors in the process. This can be leveraged to move towards a more personalized therapeutic regimen in the future

## Supplementary information

Supplemental Material Clean

## Data Availability

The code for the models and training procedures as well as result analysis will be made available through the public Github repository upon publication. Additional information and other data related to this study is available upon request to the corresponding author. Sensitive patient data is carefully and thoroughly anonymized.
