# Supplementary material for: Evaluation of OCT biomarker changes in treatment-naive neovascular AMD using a deep semantic segmentation algorithm
Source: Eye (Lond). 2024 Jul 27;38(16):3180–6. doi: 10.1038/s41433-024-03264-1 (PMC11543941; doi:10.1038/s41433-024-03264-1)
Supplement: Supplementary file 1 — Supplemental Material Clean [file 41433_2024_3264_MOESM1_ESM.docx]

Supplement:

**Data, model training and automatic OCT segmentation:**

**
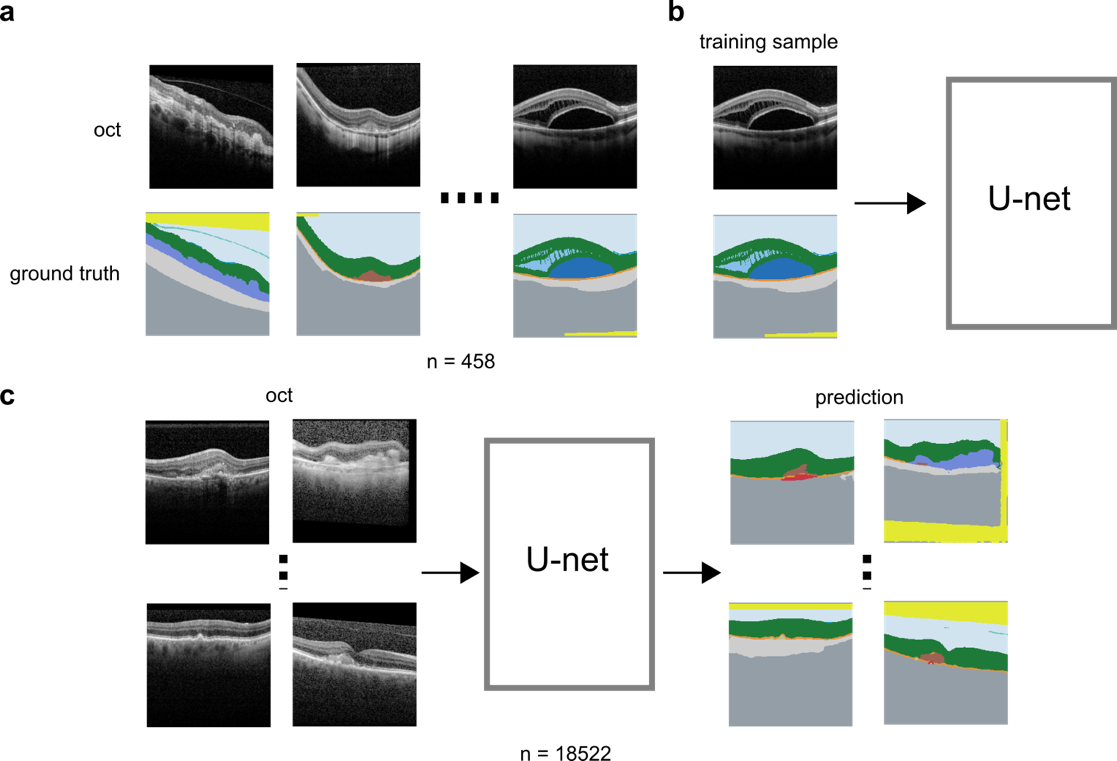
**

**
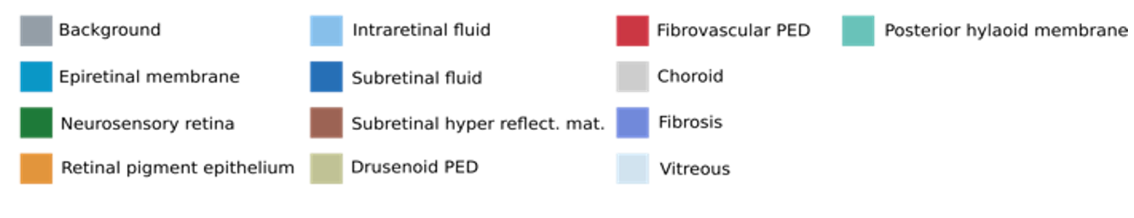
**

**Figure 3 Supplement.** *a, 458 OCT B-volumes are manually annotated for b, training U-net like deep segmentation models used for c, automatic segmentation of 18522 OCT volumes of longitudinal data.*

**
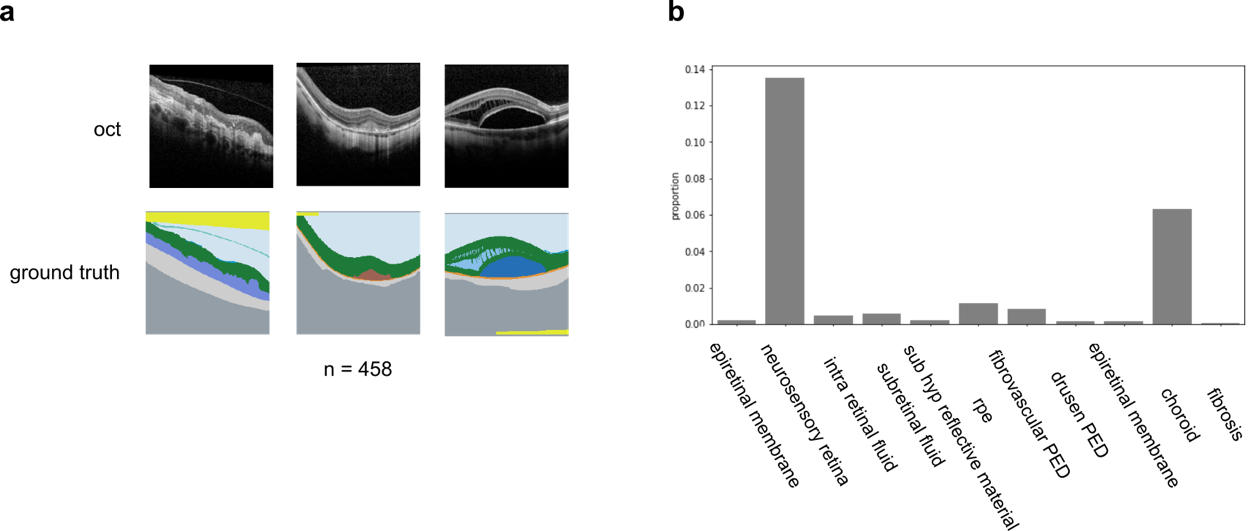

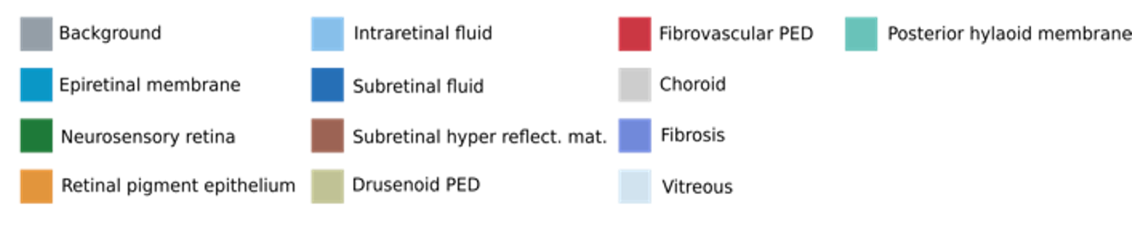
**

**Figure 4 Data Supplement.** a, three example OCT B-scans and their ground truth annotations. In b we see the proportion of each class label. Neurosensory retina and choroid dominates the labeled pixels.

**
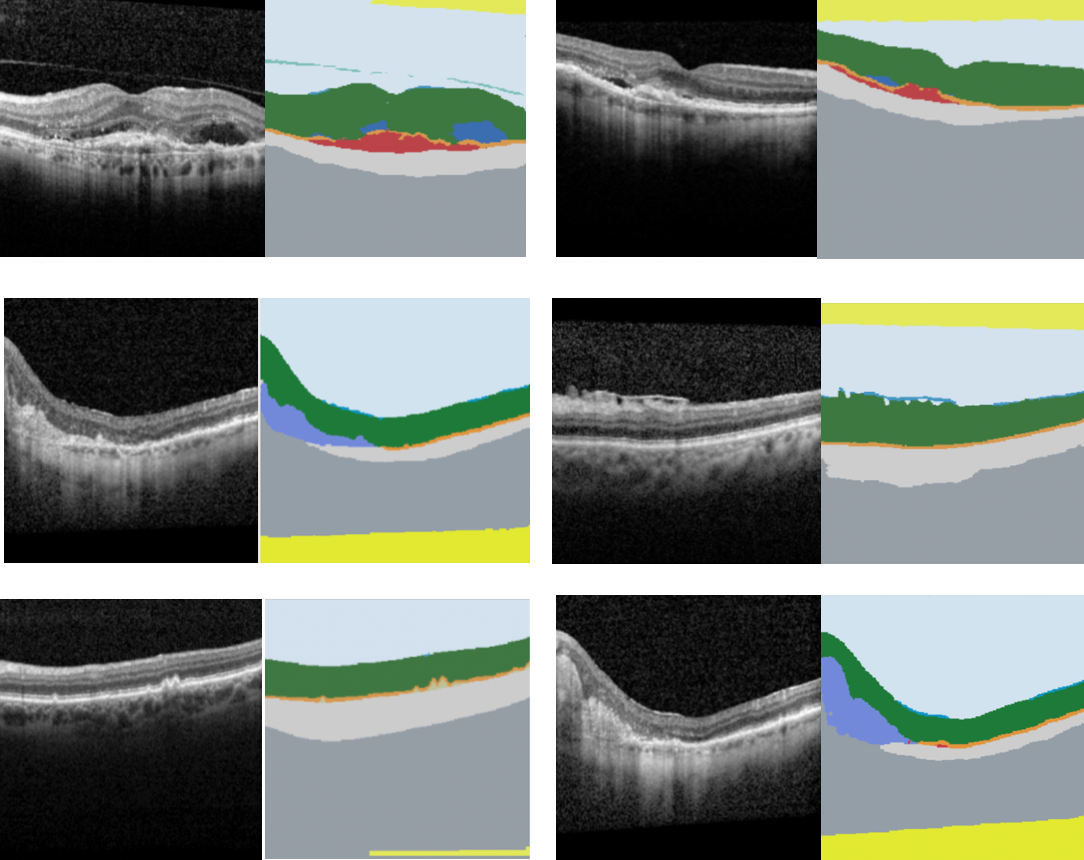
**


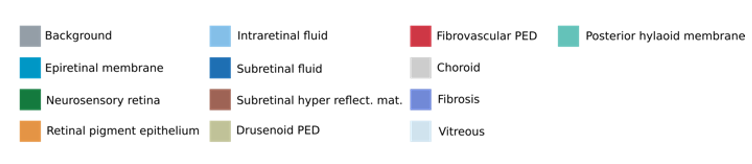


Figure 5. Examples illustrating the segmentation of various labels using the automated deep semantic segmentation algorithm.
